# Large regional variation in cardiac closure procedures to prevent ischemic stroke in Switzerland a population-based small area analysis

**DOI:** 10.1371/journal.pone.0291299

**Published:** 2024-01-02

**Authors:** Nina Stoller, Maria M. Wertli, Alan G. Haynes, Arnaud Chiolero, Nicolas Rodondi, Radoslaw Panczak, Drahomir Aujesky

**Affiliations:** 1 Department of General Internal Medicine, Inselspital, Bern University Hospital, University of Bern, Bern, Switzerland; 2 Emergency Department, Inselspital, Bern University Hospital, University of Bern, Bern, Switzerland; 3 Department of Internal Medicine, Kantonsspital Baden, Baden, Switzerland; 4 CTU Bern, University of Bern, Bern, Switzerland; 5 Institute of Primary Health Care (BIHAM), University of Bern, Bern, Switzerland; 6 School of Population and Global Health, McGill University, Montreal, Canada; 7 Population Health Laboratory (#PopHealthLab), University of Fribourg, Fribourg, Switzerland; 8 Institute of Social and Preventive Medicine, University of Bern, Bern, Switzerland; Public Library of Science, UNITED KINGDOM

## Abstract

**Background:**

Percutaneous closure of a patent foramen ovale (PFO) or the left atrial appendage (LAA) are controversial procedures to prevent stroke but often used in clinical practice. We assessed the regional variation of these interventions and explored potential determinants of such a variation.

**Methods:**

We conducted a population-based analysis using patient discharge data from all Swiss hospitals from 2013–2018. We derived hospital service areas (HSAs) using patient flows for PFO and LAA closure. We calculated age-standardized mean procedure rates and variation indices (extremal quotient [EQ] and systematic component of variation [SCV]). SCV values >5.4 indicate a high and >10 a very high variation. Because the evidence on the efficacy of PFO closure may differ in patients aged <60 years and ≥60 years, age-stratified analyses were performed. We assessed the influence of potential determinants of variation using multilevel regression models with incremental adjustment for demographics, cultural/socioeconomic, health, and supply factors.

**Results:**

Overall, 2574 PFO and 2081 LAA closures from 10 HSAs were analyzed. The fully adjusted PFO and LAA closure rates varied from 3 to 8 and from 1 to 9 procedures per 100,000 persons per year across HSAs, respectively. The regional variation was high with respect to overall PFO closures (EQ 3.0, SCV 8.3) and very high in patients aged ≥60 years (EQ 4.0, SCV 12.3). The variation in LAA closures was very high (EQ 16.2, SCV 32.1). In multivariate analysis, women had a 28% lower PFO and a 59% lower LAA closure rate than men. French/Italian language areas had a 63% lower LAA closure rate than Swiss German speaking regions and areas with a higher proportion of privately insured patients had a 86% higher LAA closure rate. After full adjustment, 44.2% of the variance in PFO closure and 30.3% in LAA closure remained unexplained.

**Conclusions:**

We found a high to very high regional variation in PFO closure and LAA closure rates within Switzerland. Several factors, including sex, language area, and insurance status, were associated with procedure rates. Overall, 30–45% of the regional procedure variation remained unexplained and most probably represents differing physician practices.

## Background

Patent foramen ovale (PFO) has a prevalence of 15–35% in the general population and may have a causal role in cryptogenic ischemic stroke and migraine [1–3]. Based on non-randomized studies showing that PFO closure may prevent recurrent stroke and migraine attacks [4, 5], percutaneous PFO closure rates increased from 1 procedure in 2000 to 899 procedures in 2010 in the UK [6, 7], and a dramatic increase in PFO closures was also seen in other countries [8]. After the publication of a randomized trial that did not support PFO closure for migraine and 3 trials that did not suggest a benefit of closure over medical therapy in terms of recurrent stroke between 2008 and 2013 [9–12], closure rates started to drop [6–8, 13]. Based on this evidence and harm related to PFO closure (procedural complications 2.6%, atrial fibrillation 4.6% [14]), earlier guidelines did not recommend routine PFO closure [15–17]. The controversy was rekindled in 2017 when 2 trials found a significant stroke reduction after PFO closure in patients aged ≤60 years [18, 19], with a subsequent rise in closure rates in Germany, France, and Italy [13].

As most atrial fibrillation associated strokes are cardioembolic and the majority of thrombi arise in the left atrial appendage (LAA) [20], percutaneous LAA closure has been developed as an alternative to anticoagulation. After 3 trials comparing LAA closure vs. anticoagulation [21–23], the effect of LAA closure on ischemic stroke still remains to be determined, with a periprocedural complication rate (e.g., pericardial effusions) of 4.2–8.7% [21–23]. Earlier guidelines stated that there is insufficient/weak evidence for the benefit of LAA closure [16, 24, 25]). In their latest guidelines, professional societies weakly recommend that LAA closure may be considered in patients with atrial fibrillation at increased risk of stroke who have a contraindication to anticoagulation [26–28].

Compared to other European countries, Switzerland and Germany had substantially higher PFO closure rates in 2018 [13]. Various factors may explain variation in PFO closure rates across countries including differing health care system related factors (e.g., access to specialists and reimbursement) [29], variation in the prevalence of PFO and PFO detection rates across countries [30, 31], and the uptake of novel interventions. Switzerland has universal health care coverage, good access to care, the same nationwide reimbursement system, and a very low regional variation in cardiovascular mortality [32] and stroke rate [33], indicating similar cardiovascular disease incidences across Swiss regions. Furthermore, the close proximity of a population with different socioeconomic and cultural factors is ideal to explore potential differences that influence treatment decisions. To date, no recent data exist on the variation in PFO/LAA closure rates within a country with a homogenous health system and universal insurance coverage. Differences in regional demographic, socioeconomic, and medical factors could provide valuable insight into the causes of procedure variation. We therefore assessed regional variation in PFO/LAA closure in Switzerland from 2013 to 2018 and examined potential determinants of variation. We hypothesized that the regional variation in PFO and LAA procedures would be low in Switzerland.

## Methods

### Data sources

We conducted a population-based, small area analysis using routinely collected patient discharge data from all Swiss public and private acute care hospitals and census data was conducted for calendar years 2013–2018. The methods used for this analysis have been previously described [34–36]. Swiss hospitals are legally obligated to provide the Swiss Federal Statistical Office with an anonymized, standardized data set for each hospital discharge. These data are then combined and centrally stored in the Swiss Hospital Discharge Masterfile. Recorded variables include patient age, sex, nationality, insurance status, the type of admission (emergency vs. elective), up to 100 procedure codes based on the Swiss Classification of Operations (CHOP, an adaptation of the U.S. ICD-9-CM volume 3 procedure classification) [37], and up to 50 diagnostic codes based on the International Classification of Diseases, 10^th^ revision, German Modification (ICD-10-GM). Further, the area of patient residence and hospital location within one of 705 Swiss MedStat regions are recorded. MedStat regions represent Swiss statistical regions based on aggregated ZIP-codes [38]. The Swiss Federal Statistical Office reviews data integrity and completeness by means of a specifically designed software [39]. Because reimbursement is based on data included in the Swiss Hospital Discharge Masterfile, annual mandatory audits are performed to ensure the accuracy of the coding in each hospital. Further, insurance companies have a mandate to oversee correctness of treatments based on the Swiss health care law. Thus, although there are not published accuracy data regarding the Swiss Hospital Discharge Masterfile, the accuracy of the data that was used for this study was most probably very high. [40].

We used Swiss National Cohort data from 2014 to determine the main language (German, French, or Italian) [41] and data from the Swiss Federal Statistical Office to determine the population density for each MedStat region. We used the average Swiss Socioeconomic Position (SSEP, version 2) as a measure of socioeconomic status. The SSEP version 2 was derived using 2012–2015 Swiss structural surveys data to rank Swiss neighborhoods based on four domains (median rent/m^2^, proportion of households with a person with no/low education, proportion of households with a person in manual/unskilled occupation, and mean crowding, all on the neighborhood level) [42]. The SSEP varies between zero (lowest) and 100 (highest) and correlates well with mortality [42]. We obtained the density of cardiologists per MedStat region for calendar year 2014 from the Swiss Medical Association. Our study was based on anonymized administrative data only and was thus, exempted from ethics committee approval according to the Swiss Human Research Act.

### Derivation of PFO and LAA closure specific hospital service areas (HSA)

Switzerland has compulsory basic health insurance coverage, with voluntary semiprivate and private insurance plans covering additional medical services. Although Swiss hospital care is primarily organized based on 26 geographic regions (the cantons) patients may utilize hospital services outside their canton of residence and the use of cantons as a unit of observation may skew procedure rates. We therefore used a fully automated method to generate reproducible general hospital service areas (HSAs) using all patient discharge data from the calendar years 2013–2016 (data that was available when the general HSAs were derived) [35]. Briefly, we identified 4,105,885 discharges of patients aged ≥18 years living in Switzerland from 155 Swiss acute care hospitals during calendar years 2013–2016. Across the 705 Swiss administrative (MedStat) regions, we identified regions that contain one or several hospitals as potential HSAs. Starting from these potential HSAs, in a centrifugal stepwise approach, we identified the geographically neighboring MedStat regions and merged them with the HSA if the majority of its residents were discharged from hospitals located in the specific HSA (plurality rule) [43]. HSAs with <50% of the patients being treated within the same HSA or <10 discharges overall were merged with the neighboring HSA which received most discharges until >50% and ≥10 discharges occurred within each HSA. This process yielded 63 general HSAs.

We then identified patient discharges with specific codes for percutaneous PFO (35.98.10–35.98.12, 35.98.19) and LAA closure (37.90.00, 37.90.10, and 37.90.99) from all Swiss acute care hospitals during calendar years 2013–2018. As PFO and LAA closures are not performed in every hospital, we analyzed patient flows for PFO and LAA procedures. Using the procedure described above, HSAs were further aggregated into 10 procedure-specific HSAs. We then drew choropleth maps of the 10 final HSAs using Geographical Information System (GIS)-compatible vector files.

### Study population

Overall, we identified 4924 patient discharges (**S1 Fig**) who underwent at least one PFO closure (N = 2574) and/or LAA closure (N = 2520). After exclusion of LAA closures not performed percutaneously (N = 439), a total of 2081 percutaneous LAA closures were analyzed. Discharges with multiple interventions (N = 170; i.e., PFO and LAA closure within the same hospital stay) were included in each procedure specific analysis.

### Measures of variation

We calculated unadjusted age- and sex-standardized PFO and LAA closure rates per 100,000 persons for each HSA using procedure counts and 2013–2018 census data for the adult Swiss population [44]. We used direct standardization with age bands of 18–49, 50–59, 60–69, 70–79 and ≥80 years. As PFO closure is recommended by guidelines in selected patients aged <60–65 years [45–47] and the procedure-related benefit was limited to patients aged < 60 years in a network meta-analysis [47], we also determined age-/sex-standardized PFO closure rates in patients aged <60 years and ≥60 years. To examine the variation in procedure rates across HSAs, we determined the extremal quotient (EQ), which is the highest, divided by the lowest procedure rate. While the EQ is an intuitive measure of variation, it is prone to distortion by extreme values [29]. We also calculated the coefficient of variation (CV), i.e., the ratio of the standard deviation of the procedures rates to the mean rate, and the systematic component of variation (SCV) [29, 48]. The SCV represents the non-random part of the variation in procedure rates while reducing the effect of extreme values [29, 48, 49]. An SCV of >5.4–10 is indicative of a high variation and an SCV of >10 of a very high variation [29, 49].

### Determinants of variation

Differences in illness incidences and socioeconomic factors are possible and legitimate causes of variation [29]. We therefore explored four regional domains that could influence procedure rates: demographics (age/sex), language region, regional socioeconomic status (median population density of persons aged ≥18 years, SSEP, percentage of discharges with semiprivate/private health insurance, and Swiss citizenship), population burden of disease, and supply factors (density of cardiologists). The language spoken by the majority of people living in a HSA was used to classify each HSA as either German or French/Italian language region as a proxy for culture [50]. We used population density as a proxy for the level of urbanization of the area a resident lived in. No information on insurance status on a population level is available in Switzerland. We thus used the insurance information in all hospitalized patients in Switzerland as a proxy for the proportion of patients that seek care in each HSA. As a proxy for the population burden of disease, we calculated age-standardized incidence rates of hip fractures (ICD 10 codes S720-22), colon (ICD 10 codes C18/19 and CHOP codes 446 or 457–58) or lung cancer (ICD 10 codes C34 and CHOP codes 323-26/329) treated surgically, acute myocardial infarctions (ICD 10 codes I21), or strokes (ICD 10 codes I63/64) for each HSA, as differences in these disease rates are likely to reflect true regional differences in burden of disease rather than differences in coding intensity or supply factors [51, 52]. The density of cardiologists was used as supply measure.

### Statistical analyses

To explore determinants of procedure rates in Switzerland, we used progressively adjusted multilevel Poisson regression with a log link to model the procedure rates in each HSA. Age was used in the bands 18 to 49, 50 to 59, 60 to 69, 70 to 79, and 80+. Model 1 included only the calendar year of the procedure. Model 2 was additionally adjusted for demographics (age/sex). Model 3 was additionally adjusted for HSA-level language region and insurance status. As population density, SSEP, and citizenship had a variance inflation factor of >5 indicating a high correlation with other variables, they were not included in the final model to avoid variance inflation and multi-collinearity of predictors [53]. Model 4 was further adjusted for HSA-level population burden of disease. Model 5 was additionally adjusted for the density of cardiologists. HSA was included as a random intercept in all models. All covariates were selected *a priori*. We depicted the variation in HSA rates as average predicted procedure rates per 100,000 persons per HSA derived from the multilevel regression models. Where rates are shown in maps, categories for the rates were chosen to be approximately equal in width.

We expressed the impact of determinants on procedure rates as incidence rate ratios (IRRs), defined as the procedure rate in the defined category (e.g., French/Italian language region) relative to the estimated procedure rate in the reference category (e.g., German language region). We also determined the percentage reduction in procedure variation across the 10 HSAs by examining the variance of the random intercept relative to model 1. We considered the residual, unexplained variation of the fully adjusted model a proxy for unwarranted variation that cannot be attributed to potential patient need [34, 36, 54]. We further assessed remaining variation in procedure rates across HSAs after full adjustment (model 5) using funnel plots. We plotted procedure rates against population size for each HSA. The mean procedure rates and the control limits of 2 and 3 standard deviations above and below the mean (95% and 99.8% confidence intervals), respectively, were calculated for all possible values for population size and used to create the funnel plot based on exact Poisson confidence intervals [55]. Statistical modeling was performed using Stata version 15.1 (StataCorp, College Station, TX). HSAs were delineated and maps drawn using the R statistical software, version 3.4.2 [56].

## Results

### Characteristics of procedure-specific HSAs and the study population

Six HSAs were located in Swiss German and four in the French/Italian-speaking part of Switzerland. The median population size aged >18 years was 136,456 persons (interquartile range [IQR] 95,883–370,190) per HSA, with a median population density of 483 persons/km^2^ (IQR 281–982). The mean proportion of residents with a (semi)private insurance was 24% (standard deviation 8) per HSA, and the median density of cardiologists 8 (IQR 7–10) per 10,000 persons.

Overall, 2574 percutaneous PFO and 2081 percutaneous LAA closure procedures were performed between 2013 and 2018. PFO closures were mainly performed in persons aged <60 years (60%) whereas most LAA closures were done in persons aged ≥70 years (80%). Patient baseline characteristics by procedure are shown in **Table 1**.

**Table 1:** Characteristics of the study population (N = 4924) undergoing PFO or LAA closure during calendar years 2013–2018.

	PFO closure (N = 2574)	LAA closure (N = 2081)
	N (%)	
Age [years]		
18–49	851 (33)	24 (1)
50–59	707 (27)	70 (3)
60–69	504 (20)	305 (15)
70–79	361 (14)	883 (42)
≥80	151 (6)	799 (38)
Sex		
Male	1470 (57)	1358 (65)
Insurance class		
General	1694 (66)	1301 (63)
(Semi)private	880 (34)	780 (37)
Swiss citizenship	2145 (83)	1851 (89)

### Variation in procedure rates across HSAs

**Fig 1:**
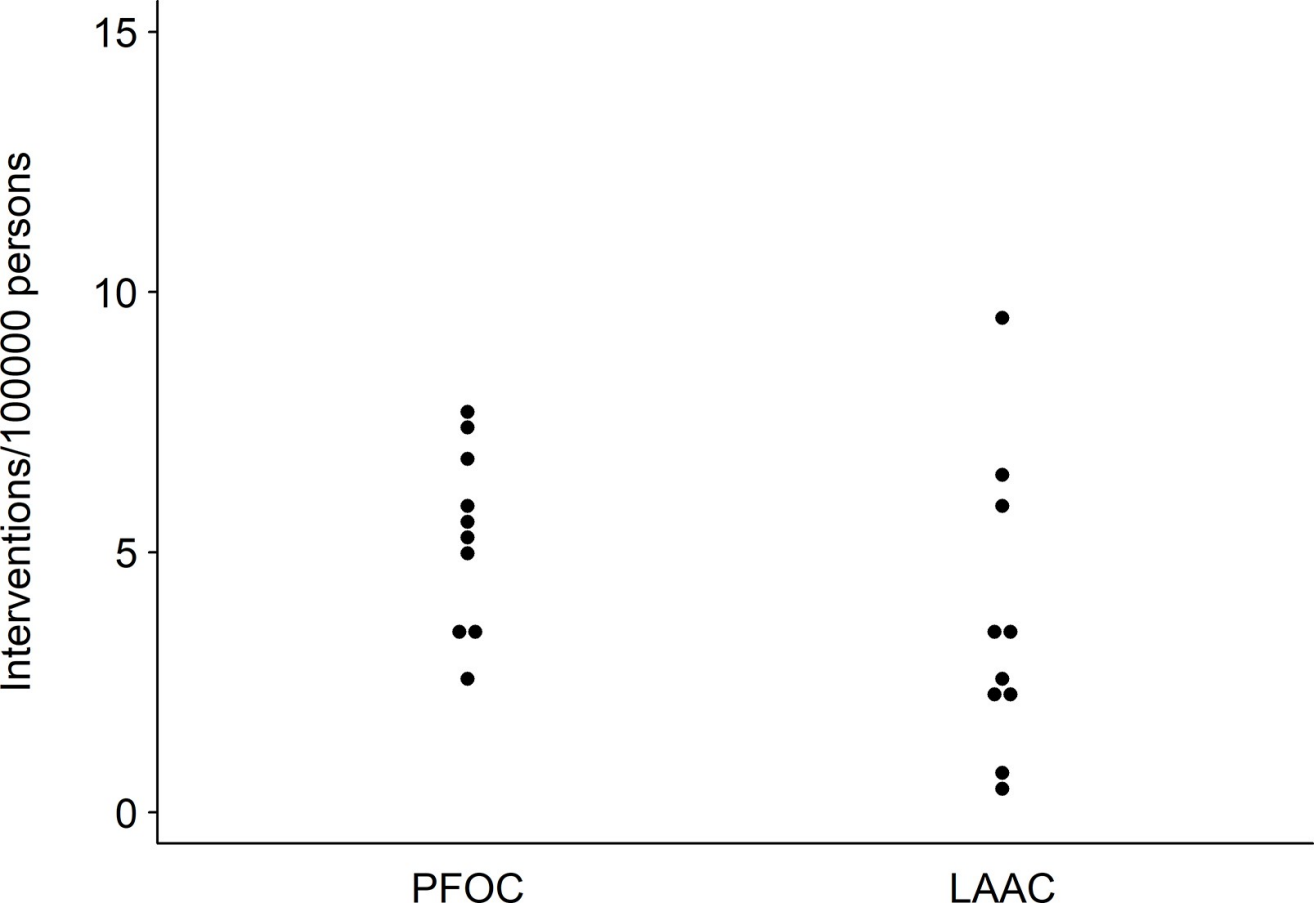
Variation in age- and sex-standardized PFO or LAA closure procedure rates per 100,000 persons across 10 Swiss hospital service areas (average rate for calendar years 2013–2018). PFOC = PFO closure. LAAC = LAA closure. Average age-/sex-standardized procedure for each HSA per 100,000 persons.

**Fig 2:**
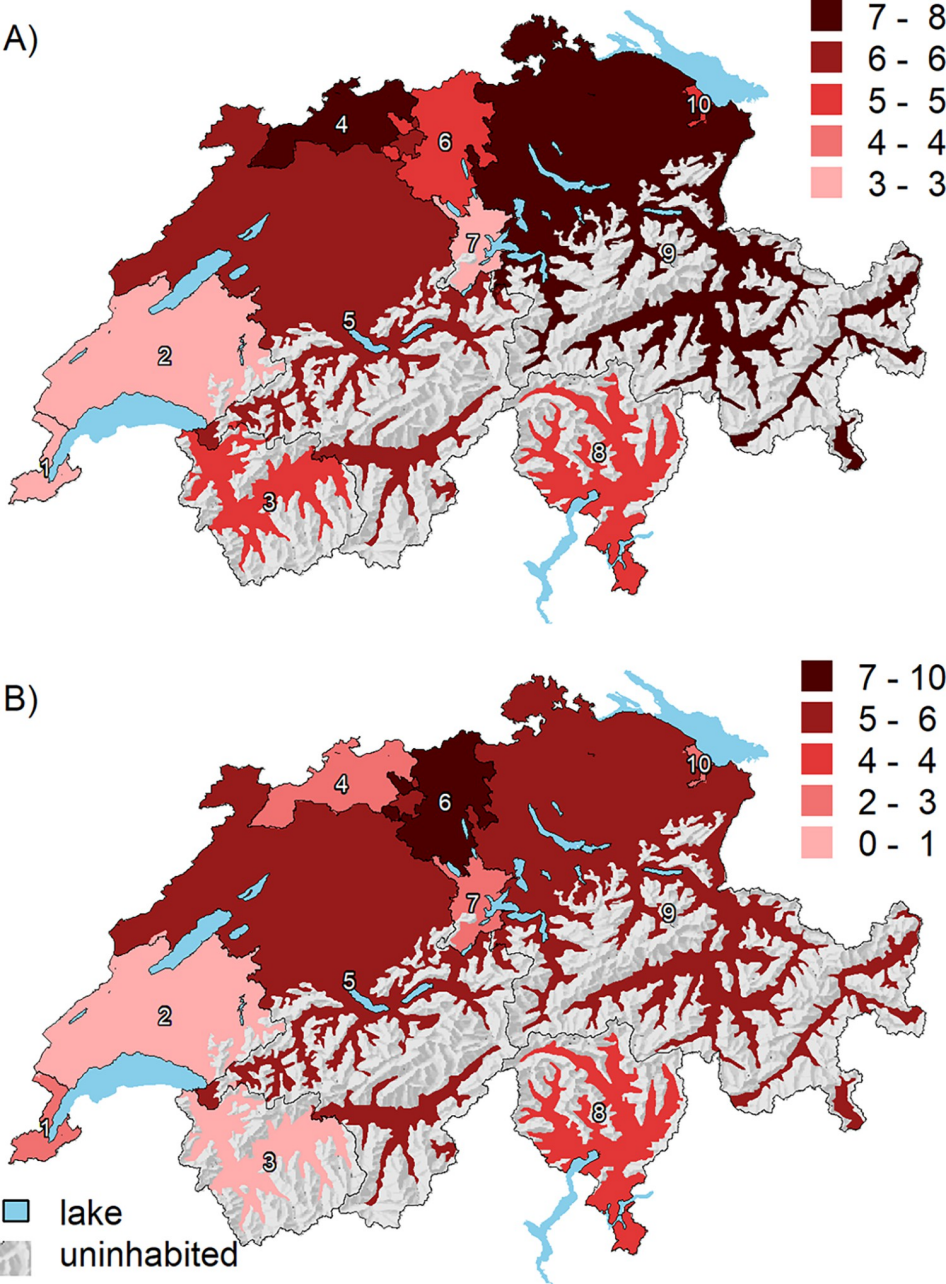
Average predicted (A) PFO or (B) LAA procedures across 10 Swiss HSAs derived from models with progressive adjustment. HSA = hospital service area. Average predicted procedures rates for each HSA are shown as red-scale categories per 100,000 persons. Adjusted for year, age/sex, language region, insurance status, burden of disease, and density of cardiologists. Shaded relief map reprinted from the Federal Office of Topography swisstopo, Switzerland https://shop.swisstopo.admin.ch/en/products/maps/overview/relief and shape files derived from postcode-level shape file used to create map of Switzerland, e.g., https://www.geocat.admin.ch/) under a CC BY license, with permission from Alexandra Frank, original copyright 2006.

The mean overall age-/sex-standardized PFO closure rate was 5 (range 3–8) per 100,000 persons (**Fig 1**), with a rate of 5 (range 2–7) in persons aged <60 years and 7 (range 3–11) per 100,000 persons in persons aged ≥60 years. Detailed age-standardized PFO closure rates for each HSA are shown in the **S1 Table.** The EQ for PFO closure was 3.0 (age <60 years: 2.9; age ≥60 years: 4.0, **Table 2**), the CV 0.3 (<60 years: 0.4; ≥60 years 0.4), and the SCV 8.3 (<60 years: 9.3; ≥60 years: 12.3). After full adjustment for procedure year, age/sex, language region, insurance status, burden of disease, and density of cardiologists, the predicted PFO closure rates varied between 3 and 8 per 100,000 persons across HSAs (**Fig 2, Panel A**), of which three had a rate above 6 (HSA number 4, 5, and 9) and three below 4 procedures per 100,000 persons (HSA 1, 2, and 7). Average predicted PFO closure procedure rates in patients aged <60 and ≥60 years after full adjustment are shown in **S2 Fig**.

**Table 2:** Variation in procedure rates across HSAs.

	PFO closure			LAA closure
	Overall	<60 years	≥60 years	Overall
EQ	3.0	2.9	4.0	16.2
CV	0.3	0.4	0.4	0.7
SCV	8.3	9.3	12.3	32.1

Abbreviations: EQ = extremal quotient; CV = coefficient of variation; SCV = systematic component of variation

The mean age-/sex-standardized rate for LAA closure was 4 (1–9) per 100,000 persons (**Fig 1**). The EQ was 16.2 (**Table 2**), the CV 0.7, and the SCV 32.1. After full adjustment, the predicted LAA closure rates varied between 1 and 9 per 100,000 persons across HSAs (**Fig 2, Panel B**).

### Determinants of variation in procedure rates

Between 2013 and 2018, procedure rates remained stable (**Table 3**). Patients aged <50 and >79 years had lower PFO closure rates than patients aged 50–79 years. Compared to persons aged 60–69 years, persons aged ≥80 years had a 6.4-fold higher LAA closure rate (IRR 6.41, 95% CI 5.62–7.32). Women were less likely to undergo procedures than men (PFO closure IRR 0.72, 95% CI 0.66–0.78; LAA closure IRR 0.41, 95% CI 0.37–0.45). Residence in a French/Italian language region was associated with 63% lower LAA closure rates than in Swiss German regions (IRR 0.37, 95% CI 0.20–0.69), whereas a 10% higher regional proportion of (semi)private insurance coverage was associated with an 86% increase in LAA closure rates (IRR 1.86, 95% CI 1.18–2.92). Neither burden of disease nor density of cardiologists had a relationship with PFO and LAA closure. Compared to the year-adjusted model, additional adjustment for demographics, language region, and insurance status explained most of the variance in closure procedures (PFO 37.4% and LAA closure 46.2%) (model 3, **Table 4**). Additional adjustment for burden of disease and density of cardiologists (model 5) explained only 10.3% of the variance in PFO and 18.0% of the variance in LAA closures. Overall, 44.2% of the variance in PFO (<60 years: 80.1%; ≥60 years: 44.2%) and 30.3% of the variance in LAA closure rates remained unexplained.

**Table 3:** Determinants of variation in the incidence rates of closure procedures across 10 Swiss HSAs.

		PFO closure	LAA closure
		Incidence rate ratio (95% CI)*	
Year (per year)		0.99 (0.95–1.03)	1.04 (0.99–1.09)
Age	18–49	**0.42 (0.38–0.47)**	**0.02 (0.02–0.03)**
	50–59	1.04 (0.93–1.17)	**0.17 (0.13–0.22)**
	60–69	Reference	Reference
	70–79	1.00 (0.87–1.15)	**4.14 (3.63–4.71)**
	≥80	**0.68 (0.56–0.81)**	**6.41 (5.62–7.32)**
Sex	Male	Reference	Reference
	**Female**	**0.72 (0.66–0.78)**	**0.41 (0.37–0.45)**
Language region	German	Reference	Reference
	**French/Italian**	0.74 (0.53–1.02)	**0.37 (0.20–0.69)**
(Semi)private insurance (per 10% change)		0.85 (0.67–1.08)	**1.86 (1.18–2.92)**
Burden of disease (per 1 per 1000)§		1.70 (0.41–7.06)	3.27 (0.53–20.23)
Density of cardiologists (per 1 per 10,000)#		1.00 (0.96–1.05)	0.92 (0.83–1.01)

Abbreviations: CI = confidence interval. Results in **bold** indicate a statistically significant effect.

*Procedure rate in the defined category relative to the procedure rate in the reference category. For instance, an incidence rate ratio of 0.72 indicates a 28% lower PFO closure rate in women than in men.

§Burden of disease represents the sum of age-standardized incidence rates of hip fracture, colon or lung cancer treated surgically, acute myocardial infarction, and stroke. The IRR is the increase (decrease) in procedure rates when the regional burden of disease increases by 1 comorbidity per 1000 persons.

#The IRR is the increase (decrease) in procedure rates when the number of cardiologists increases by 1 per 10,000 persons.

**Table 4:** Remaining variance after incremental adjustment*.

	Model 2§	Model 3#	Model 4†	Model 5‡
	% of remaining variance			
PFO closure	91.9	54.5	44.3	44.2
<60 years	93.2	67.8	80.0	80.1
≥60 years	98.6	51.8	44.1	44.2
LAA closure	94.5	48.3	40.9	30.3

*Reference: model 1 (adjustment for procedure year only)

§adjusted for age and sex

#additionally adjusted for language region and (semi)private insurance

†additionally adjusted for burden of disease

‡additionally adjusted for density of cardiologists

When plotting procedure rates against population size, several HSAs had rates above or below the outer control limits of 3 standard deviations, indicating unusually high (HSAs 5, 6, and 9) or low procedure rates (HSA 2 and 3) (**S3 Fig**). HSAs with high rates were located in the central area of Switzerland, and tended to have a higher proportion of (semi)private insurance coverage but a similar density of cardiologists and disease burden (**S2 Table**).

## Discussion

Our study shows a high to very high regional variation in PFO and LAA closure rates across Swiss HSAs. Women had significantly lower procedures rates than men for both procedures. Swiss German language regions and regions with a higher proportion of (semi)privately insured persons had higher LAA rates. Disease burden and the density of cardiologists did not affect procedure rates. Overall, 44% of the variation in PFO and 30% of the variation in LAA closure rates remained unexplained.

The average Swiss, German, and Italian PFO closure rates (average 5 per 100,000 persons) appears to be substantially higher than in France (2.9 per 100,000) and the UK (0.5 per 100,000) [13]. While differing procedure rates across countries could be caused by health care system related differences [29] or differences in the prevalence of PFO [30] and the PFO detection rates, the high regional variation in PFO closure within Switzerland, a country with a homogenous health care system and good access to care, is unclear. With a low variation in stroke rates across Switzerland [33] and assuming similar patient preferences across Swiss regions, the variation is most likely partially due to differing local practice patterns [57]. Previous studies on factors influencing the choice of therapy in patients with coronary artery disease showed, that many factors such as educational level, patient functioning, and gender influence the treatment choice [58]. Such factors may also influence the decision to search for open PFO and a treatment decision. A survey conducted in 79 hospitals from 34 countries showed a substantial variation in the intensity with which PFO is sought (e.g., by using more sensitive transesophageal rather than transthoracic echocardiography as the initial diagnostic test for PFO) and other potential causes for stroke are excluded (e.g., single electrocardiogram vs. Holter monitoring to detect atrial fibrillation) after cryptogenic stroke [59]. Similarly, a survey among 120 specialists reported major differences in their perception about PFO as the stroke etiology [60].

Variation in local practice patters may be a direct consequence of the ongoing controversy whether PFO is causally related with cryptogenic stroke or just an innocent bystander as well as differing guideline recommendations. While the most recent guidelines support the use of PFO closure in carefully selected patients aged <60–65 years with a recent ischemic stroke attributed to a PFO and encourage shared decision-making [45–47, 61], they differ in several aspects, including the duration of cardiac monitoring to exclude atrial fibrillation as the stroke etiology, and the level of evidence (A to C) for PFO closure. PFO closure for the sake of migraine is usually not recommended outside clinical trials [62]. Moreover, cardiologists’ decision making may be more shaped by individual, interpersonal, organizational, and financial influences and only to a limited extent by guidelines [63]. Overall, the adherence to recommendations for most management strategies for cryptogenic stroke and PFO appears to be variable according to an international survey [59]. In our study, 40% of the PFO closures were done in individuals older than ≥60 years, with a particularly high variation in this age group across HSAs (SCV 12.3). Our finding that women had a 28% lower PFO closure rate than men could be due to the fact that women develop stroke at a later age than men and thus may less often be considered for PFO closure [64].

Given the limited evidence for benefit, the risk of periprocedural complications, and the lack of long-term outcome data [65], percutaneous LAA closure is even more controversial. In their latest guidelines, professional societies weakly recommend LAA closure in patients with atrial fibrillation at increased risk of stroke who have a contraindication to anticoagulation [26–28]. International surveys suggest that cardiologists’ perception of LAA closure in terms of benefit/risk and indications for LAA closure vary widely [66, 67].

We observed higher LAA closure rates with increasing age in our study. This finding is explained by the higher prevalence of persons with atrial fibrillation who have an increased bleeding risk in the elderly population [68, 69]. Further, older age was associated with an increased risk for periprocedural complications and mortality [70]. Also, women had a 59% lower likelihood to undergo LAA closure. Although women have a lower age-adjusted incidence of atrial fibrillation than men [68], women with atrial fibrillation have a 31% higher stroke risk and a higher mortality than men [71, 72]. It is established that women are less likely to undergo electrical cardioversion and catheter ablation of atrial fibrillation than men [73, 74] and had a substantially lower implantation rate in cardiac implantable electronic devices [75], raising the possibility of referral bias against these interventions in women [76]. Thus, our finding of substantially lower LAA closure rates in women warrants further studies to elucidate the underlying reasons for this disparity.

French/Italian speaking areas had a 63% lower LAA closure rate than Swiss German regions, possibly due to more conservative physician practice styles in these areas. We previously found lower rates of other preference-sensitive interventions in the French/Italian speaking regions of Switzerland, including cardiac implantable electronic devices, vertebroplasty, hysterectomy, and joint arthroplasty [34, 36, 54, 75]. We also found an 86% higher LAA rate in regions with a higher prevalence of (semi)private insurance, raising the question whether additional insurance (which results in higher physician fees) may induce overtreatment.

Overall, a remarkably high proportion of the regional variation in PFO/LAA closures (30–80%) remained unexplained by demographic and socioeconomic factors, disease burden, and the density of cardiologists. Patient-level factors are highly unlikely to explain differences in procedure rates in neighboring areas. For example, HSAs 7 and 9 are neighboring regions with a high and a low PFO / LAA closure rate, respectively. Patient’s characteristics and disease prevalence and severity are unlikely to differ between HSAs with such close geographical proximity. It has been shown that practice variation in preference-sensitive procedures, such as PFO and LAA closure procedures, is mainly explained by individual physicians’ beliefs about the indication and efficacy of the procedure and not by patient need or preferences [29, 77]. Thus, unexplained variation in PFO/LAA closure is more likely to be due to local cardiologists’ attitudes and socioecological influences rather than guideline recommendations [63].

Our study has several limitations. First, we did not have clinical data, including procedure indication, severity of the disease, and relevant comorbidities. Therefore, we could not verify the appropriateness of procedure performance. Although we had no information on the prevalence of atrial fibrillation, we adjusted for age-standardized incidence rates of hip fractures, colon/lung cancer treated surgically, acute myocardial infarctions, or strokes for each hospital service areas as proxy for the population burden of disease. Regional differences in the rates, which practically always require hospitalization, are likely to reflect true regional differences in burden of disease. Thus, we adjusted for the incidence of stroke rates in our models. Second, we had no information about other potential drivers of regional variation in PFO/LAA closure, including differences in patient preferences, physician attitudes and technical expertise, and hospitals in which procedures are performed. In particular, we had no information on the number of interventional cardiologists and the experience of the operators that perform the procedures and the volume of procedures that are performed in each center. These factors should be further explored. Finally, adjustment for ecological variables on a population level (i.e., age, language, insurance, etc.) carries a risk of ecological fallacy by drawing conclusions about the behavior of individuals based on population parameters [78].

In conclusion, we found a high regional variation in PFO and a very high variation in LAA closure among Swiss HSAs. Procedure rates were significantly lower in women and also depended on language region and insurance status. A substantial part of the regional variation in both procedures remained unexplained and is most likely due to differing practices of local physicians rather than to patient need or preferences.

## Supporting information

S1 Fig Study flow chart. (TIF)

S2 Fig Average predicted PFO closure procedure rates across 10 Swiss HSAs derived from models with progressive adjustment. Panel A: Patients aged <60 years, Panel B: Patients aged ≥60 years. Abbreviations: uninhab. = uninhabited area; HSA = hospital service area. Average predicted PFO closure procedure rates for each HSA are shown as red-scale categories per 100,000 persons. Adjusted for age/sex, language region, insurance status, burden of disease, and the density of cardiologists. Shaded relief map reprinted from the Federal Office of Topography swisstopo, Switzerland https://shop.swisstopo.admin.ch/en/products/maps/overview/relief and shape files derived from postcode-level shape file used to create map of Switzerland, e.g., https://www.geocat.admin.ch/) under a CC BY license, with permission from Alexandra Frank, original copyright 2006.
(TIF)

S3 Fig Variation in fully adjusted PFO and LAA rates (model 5) across 10 HSAs by funnel plot analysis. The straight line indicates the mean procedure rate; the inner and outer control limits represent 2 and 3 standard deviations above/below the mean, respectively (i.e., 95% and 99.8% of data, respectively). Thus of 10 HSAs, <0.1 could be expected to lie beyond the outer control limit by chance; 0.5 HSAs could be expected to lie above or below the inner control limit by chance. HSAs with procedure rates that fall out of the outer control limits are tagged by their given HSA-number indicating potential over- and under-treatment in the specific HSAs respectively (consult S2 Table for details).
(TIF)

S1 Table PFO and LAA closure rates for each has. *adjusted for year, age, sex, language region, (semi)private insurance, burden of disease and density of cardiologists); rates per 100,000 persons; 95% confidence intervals in parentheses.
(DOCX)

S2 Table Characteristics of HSAs with PFO/LAA rates outside the funnel plot control lines. Abbreviations: HSA = hospital service area. *adjusted for year, age, sex, language region, (semi)private insurance, burden of disease and density of cardiologists), 95% confidence intervals in parentheses. §language region within Switzerland, i.e. either German or French/Italian speaking region. #burden of disease represents the sum of age-standardized incidence rates for the following comorbidities: hip fracture, colon or lung cancer treated surgically, acute myocardial infarction, and stroke. †HSA with PFO procedure above or below the 99.8% confidence intervals. ‡HSA with LAA procedure above or below the 99.8% confidence intervals.
(DOCX)

S1 File (PDF)
