# Supplementary material for: Large regional variation in cardiac closure procedures to prevent ischemic stroke in Switzerland a population-based small area analysis
Source: PLoS One. 2024 Jan 2;19(1):e0291299. doi: 10.1371/journal.pone.0291299 (PMC10760725; doi:10.1371/journal.pone.0291299)
Supplement: S1 Table — *adjusted for year, age, sex, language region, (semi)private insurance, burden of disease and density of cardiologists); rates per 100,000 persons; 95% confidence intervals in parentheses. (DOCX) [file pone.0291299.s004.docx]

**S1 Table. PFO and LAA closure rates for each HSA**

| **HSA** | **PFO rates** | | | **LAA rates** | | |
| --- | --- | --- | --- | --- | --- | --- |
|  | **Crude** | **Standardized (age/sex)** | **Fully adjusted*** | **Crude** | **Standardized (age/sex)** | **Fully adjusted*** |
| 6 | 5.8 | 5.7 | 5.8 (4.2 - 7.9) | 9.1 | 9.4 | 9.0 (5.3 - 15.2) |
| 5 | 6.9 | 6.7 | 6.9 (5.3 - 9.1) | 6.5 | 6.0 | 6.5 (4.0 - 10.7) |
| 4 | 7.2 | 7.3 | 7.1 (5.3 - 9.4) | 2.8 | 2.5 | 2.9 (1.8 - 4.7) |
| 2 | 3.3 | 3.5 | 3.4 (2.5 - 4.7) | 0.7 | 0.8 | 0.8 (0.4 - 1.4) |
| 1 | 3.2 | 3.3 | 3.2 (2.0 - 5.1) | 2.2 | 2.4 | 2.2 (0.9 - 5.3) |
| 7 | 2.6 | 2.6 | 3.1 (2.1 - 4.7) | 3.5 | 3.5 | 3.7 (2.0 - 7.0) |
| 10 | 4.7 | 4.8 | 5.2 (2.7 - 10.0) | 2.5 | 2.4 | 2.2 (0.7 - 7.0) |
| 8 | 5.9 | 6.0 | 5.7 (3.9 - 8.4) | 4.4 | 3.6 | 4.2 (2.3 - 7.9) |
| 3 | 5.3 | 5.3 | 5.3 (3.3 - 8.6) | 0.6 | 0.6 | 0.6 (0.2 - 1.7) |
| 9 | 7.7 | 7.6 | 7.6 (5.9 - 9.9) | 6.3 | 6.4 | 6.3 (4.0 - 9.9) |

*adjusted for year, age, sex, language region, (semi)private insurance, burden of disease and density of cardiologists); rates per 100,000 persons; 95% confidence intervals in parentheses.
