# Supplementary material for: Large regional variation in cardiac closure procedures to prevent ischemic stroke in Switzerland a population-based small area analysis
Source: PLoS One. 2024 Jan 2;19(1):e0291299. doi: 10.1371/journal.pone.0291299 (PMC10760725; doi:10.1371/journal.pone.0291299)
Supplement: S2 Table — Abbreviations: HSA = hospital service area. *adjusted for year, age, sex, language region, (semi)private insurance, burden of disease and density of cardiologists), 95% confidence intervals in parentheses. §language region within Switzerland, i.e. either German or French/Italian speaking region. #burden of disease represents the sum of age-standardized incidence rates for the following comorbidities: hip fracture, colon or lung cancer treated surgically, acute myocardial infarction, and stroke. †HSA with PFO procedure above or below the 99.8% confidence intervals. ‡HSA with LAA procedure above or below the 99.8% confidence intervals. (DOCX) [file pone.0291299.s005.docx]

**S2 Table. Characteristics of HSAs with PFO/LAA rates outside the funnel plot control lines**

| **HSA** | **Fully adjusted PFO rate**  **(100,000 persons/year)*** | **Fully adjusted LAA rate**  **(100,000 persons/year)*** | **Language**  **region§** | **(Semi) private insurance (%)** | **Burden of disease (per 1000 persons)#** | **Density of cardiologists (per 10,000 persons)** |
| --- | --- | --- | --- | --- | --- | --- |
| 9†‡ | 7.6 (5.9 - 9.9) | 6.3 (4.0 - 9.9) | German | 27.76 | 1.03 | 7.25 |
| 5† | 6.9 (5.3 - 9.1) | 6.5 (4.0 - 10.7) | German | 18.78 | 1.09 | 7.38 |
| 6† | 5.8 (4.2 - 7.9) | 9.0 (5.3 - 15.2) | German | 23.68 | 1.07 | 5.49 |
| 3† | 5.3 (3.3 - 8.6) | 0.6 (0.2 - 1.7) | Romance | 8.64 | 1.00 | 7.66 |
| 2† | 3.4 (2.5 - 4.7) | 0.8 (0.4 - 1.4) | Romance | 22.20 | 1.03 | 8.12 |

Abbreviations: HSA = hospital service area

*adjusted for year, age, sex, language region, (semi)private insurance, burden of disease and density of cardiologists), 95% confidence intervals in parentheses.

§language region within Switzerland, i.e. either German or French/Italian speaking region.

#burden of disease represents the sum of age-standardized incidence rates for the following comorbidities: hip fracture, colon or lung cancer treated surgically, acute myocardial infarction, and stroke.

†HSA with PFO procedure above or below the 99.8% confidence intervals.

‡HSA with LAA procedure above or below the 99.8% confidence intervals.
